# Orthorexia nervosa: an investigation in the context of personality, body image, and physical activity

**DOI:** 10.3389/fpsyg.2025.1672773

**Published:** 2025-11-07

**Authors:** Halil İbrahim Genç, Sezgin Hepsert, Tuncay Öktem, Tuncay Kiratli, Yavuz Yildirim, Özgecan İlgin

**Affiliations:** 1 Faculty of Tourism, Sakarya University of Applied Sciences, Sakarya, Türkiye; 2 Independent Researcher, Elazığ, Türkiye; 3 Faculty of Sport Sciences, Bayburt University, Bayburt, Türkiye; 4 Independent Researcher, Sakarya, Türkiye; 5 Graduate School of Education, Department of Recreation, Sakarya University of Applied Sciences, Sakarya, Türkiye; 6 Independent Researcher, İzmir, Türkiye

**Keywords:** orthorexia nervosa, personality, body image, physical activity, big five personality model

## Abstract

This study aimed to examine the relationship between orthorexia nervosa and body image, physical activity level, and the Big Five personality traits among non-clinical adult individuals. Based on the relational survey model, the research sample consisted of 350 undergraduate students (205 females, 145 males) enrolled in various departments at a public university. A convenience sampling method was used, and participants’ ages ranged from 18 to 30 years (*M* = 20.64, SD = 2.07). Data were collected using a personal information form, the ORTO-15 Scale, the Big Five Personality Test (short version), the Body Appreciation Scale, and the International Physical Activity Questionnaire. Pearson correlation and regression analyses were conducted. The findings revealed that conscientiousness, openness to experience, and body image were significantly associated with orthorexic tendencies. As levels of conscientiousness and openness to experience increased, behaviors related to orthorexia nervosa also tended to increase. Similarly, individuals with higher body image satisfaction exhibited more pronounced orthorexic tendencies. These results suggest that individual differences may play a determining role not only in healthy lifestyle choices but also in the potential development of these choices into pathological behaviors.

## 1 Introduction

Nutrition is one of the basic physiological needs of human life, but in modern societies it has begun to take on increasingly complex psychological and sociocultural meanings. In recent years, individuals’ eating behaviors have transcended the mere fulfillment of biological needs, becoming an extension of identity construction, body image control, and the ideology of healthy living. This transformation can sometimes lead to pathological levels of rigid and obsessive behaviors in the pursuit of healthy eating. Especially in contemporary societies, the rising “clean eating” trend is associated with obsessive food selectivity and behavioral patterns leading to social isolation (Hsia, 2023; Catapano et al., 2023).

The concepts of healthy living and nutrition are increasingly becoming the focus of various scientific disciplines and can affect an individual’s psychological, social, and biological health in multiple ways (Niedzielski and Kaźmierczak-Wojtaś, 2021; Strahler et al., 2020). However, the lack of scientific consensus regarding the protective or harmful nature of food substances can create uncertainty and anxiety among individuals (Fardet and Boirie, 2014). In this context, the concept of Orthorexia Nervosa (ON), introduced to the literature by Bratman (1997), is defined by the rigid rules, intense need for control, and obsessive behaviors that individuals develop in the process of consuming foods believed to be healthy (Cena et al., 2019; Łucka et al., 2019).

It has been shown that ON is associated with inadequate nutrient intake at both the macro and micro levels, social dysfunction, and anxiety even in non-clinical individuals (Dolapoglu et al., 2023). It has also been suggested that it presents a spectrum-like structure and that behaviors that begin with healthy eating motivation can become pathological over time (Bratman, 2017). However, it is not yet recognized as an official diagnostic category in either the DSM-V or ICD-11 classifications. This situation indicates that empirical gaps persist in the determination of diagnostic criteria and that international standardization has not been achieved (Moroze et al., 2015; Dunn and Bratman, 2016).

Recent meta-analyses indicate that the prevalence of orthorexia nervosa (ON) is particularly high among athletes and individuals focused on body composition. For example, in a study by Hafstad et al. (2023), the prevalence of ON among individuals who exercise was found to be between 43.2 and 66.8%, with an overall prevalence rate of 55.3% (López-Gil et al., 2023; Hafstad et al., 2023). Another study indicated that ON symptoms ranged from 23.5 to 31.6% in the general population and rose to 34.5% among athletes. These findings reveal that ON has significantly varying prevalence rates across different populations and carries a higher risk, particularly among athletes and individuals focused on body composition (López-Gil et al., 2023). Although orthorexia nervosa is prevalent in different sample groups, the number of studies examining its cognitive, behavioral, and personality-based components is limited. In particular, the relationships between body image, physical activity levels, and personality traits are inconsistent and show contextual differences (Chaki et al., 2013; Dunn and Bratman, 2016). The current literature suggests that high levels of perfectionism, neuroticism, and conscientiousness may be associated with increased anxiety about body image and intense exercise behaviors. These findings suggest that certain personality profiles may predispose individuals to increased body awareness and health-focused behaviors, thereby contributing to orthorexia tendencies. This summary provides a broader context for understanding the psychological determinants of eating behaviors and physical activity patterns. The psychodynamic or personality-based dimensions of ON have not been sufficiently elucidated; studies examining it within the framework of the Big Five personality traits are particularly scarce (Golestanbagh et al., 2021).

In this context, it is clear that ON exhibits a multidimensional structure that cannot be reduced to dietary preferences alone. Therefore, it is important to evaluate this concept in conjunction with personality tendencies, body image, and behavioral patterns in order to understand the disorder. The aim of this study is to identify orthorexic tendencies in a population consisting of young, educated students and to examine the relationship between these tendencies and body image, physical activity level, and five major personality traits. In this context, the study investigated how obsessive behaviors focused on healthy eating are linked to personality-based tendencies and whether body image disturbances contribute to this risk structure. Because our sample consisted of university students and individuals in health-related disciplines may be at higher risk for orthorexia, the generalizability of the findings to the general adult population is limited. Furthermore, the study did not include preliminary screening or control questions for participants regarding the presence of eating disorders or other psychiatric disorders; this was considered a methodological limitation. The research aims to provide theoretical contributions to diagnostic processes by better understanding the psychological origins of this disorder.

## 2 Method

### 2.1 Research model

The study utilized the “correlational survey” model. The correlational survey model is defined as a scientific approach that aims to reveal the relationship or effect between two different quantitative variables (Fraenkel et al., 2012).

### 2.2 Sample size

The sample size was determined by applying a power analysis to the study. The minimum sample size required was calculated using the effect sizes recommended by Cohen (1992) when calculating the effect size. As a result of the *a priori* power analysis, the required sample size was determined to be a total of 319 individuals. Gignac and Szodorai (2016) recommended increasing the sample size to account for the possibility of attrition. Accordingly, a 10% increase was applied, resulting in a sample size of 350 individuals. When calculating the sample size, the following parameters were used: Correlation: Bivariate normal model, p H1 = 0.20, *α* err prob. = 0.05, Power (1-*β* err prob) = 0.95, p H0 = 0, Tail(s) = two. The *a priori* power analysis used in calculating the sample size was performed using the G*Power 3.1.9.7 program (Franz Faul, University of Kiel, Germany).

### 2.3 Research group

A total of 350 students, 205 females and 145 males, enrolled in different undergraduate programs at a state university participated in the study. Convenience sampling (Karagöz, 2017) was used in the sample selection, and the participants’ ages ranged from 18 to 30 (X_age_ = 20.64 ± 2.07).

Table 1 shows that 58.6% (*n* = 205) of participants were female and 41.4% (*n* = 145) were male. When the distribution by department is examined, it is determined that 42.6% (*n* = 149) of the participants are studying Guidance, 21.4% (*n* = 75) are studying Gastronomy, 18.6% (*n* = 65) are studying Business Administration, and 17.4% (*n* = 61) are studying Recreation. The average age of the participants was calculated as 20.64 (SD = 2.07).

**Table 1:** Descriptive information about participants.

	*n*	%
Gender		
Female	205	58.6
Male	145	41.4
Department		
Gastronomy	75	21.4
Guidance	149	42.6
Business	65	18.6
Recreation	61	17.4
Age	M = 20.64	SD = 2.07
Total	350	100.0

### 2.4 Data collection tools

#### 2.4.1 Personal information form

The personal information form prepared by researchers consists of questions aimed at learning personal information such as gender, age, and department of individuals participating in the research.

#### 2.4.2 Orthorexia-15 scale

The ORTO-15 scale developed by Donini et al. (2005) and colleagues was created based on Bratman’s 10-item short questionnaire on Orthorexia Nervosa. Some items were removed from the scale and new statements were added. Initially applied to Latin individuals in Italy, this self-assessment scale aims to evaluate individuals’ obsessive behaviors related to healthy eating. The scale consists of a total of 15 items and is structured in a 4-point Likert format (always, often, sometimes, never). The items measure individuals’ obsessive attitudes toward selecting, purchasing, preparing, and consuming foods they define as healthy. The scale was adapted into Turkish by Arusoğlu (2006), and as a result of validity and reliability studies, the cutoff score was determined to be 33. Individuals scoring below this threshold score show orthorexic tendencies, while as the score increases, eating behavior approaches normal limits. Therefore, ORTO-15 only has a structure that measures orthorexia risk.

#### 2.4.3 Big five-50 personality test

In this study, the Big Five-50 Personality Test, adapted into Turkish by Tatar (2017), was used to determine students’ personality traits. The scale measures the levels of the five basic dimensions of personality: extraversion, agreeableness, conscientiousness, emotional stability, and openness to experience. The scale consists of a total of 50 items, each rated on a five-point Likert format (“Not at all appropriate,” “Not appropriate,” “Undecided/Average,” “Somewhat appropriate,” and “Very appropriate”). In the adaptation study, the internal consistency coefficients for the scale’s subscales ranged from 0.67 to 0.79. High scores on the scale indicate that the individual exhibits more pronounced characteristics in the relevant personality dimension (Tatar, 2017). Furthermore, this explanation also demonstrates the potential effects of personality dimensions on individuals’ behaviors and tendencies.

#### 2.4.4 Body appreciation scale

The Body Appreciation Scale developed by Tylka and Wood-Barcalow (2015) was used to assess participants’ body images. The scale consists of 10 items and is rated on a 5-point Likert format (1 = never, 5 = always). The body image score is calculated based on the total of all items on the scale, and there are no reverse-scored items. Adapted into Turkish by Anlı et al. (2024) and colleagues, high scores on this scale indicate that the individual exhibits a high level of body appreciation. Thus, the scores obtained on the scale reflect individuals’ ways of perceiving and evaluating their own bodies and help to understand the relationship between body image levels and different behavioral and psychological tendencies.

#### 2.4.5 International physical activity questionnaire short form

Participants’ physical activity levels were determined using the International Physical Activity Questionnaire (IPAQ). The validity and reliability study for adapting the questionnaire to Turkey was conducted by Öztürk (2005). In this study, a short form was used that could be completed by individuals using a self-report method and covered the “last seven days.” The short form, consisting of seven items, provides information on the time individuals spend sitting, as well as the time spent walking and engaging in moderate- and high-intensity physical activities. The total physical activity score obtained from the short form is calculated by multiplying the time (minutes) and frequency (days) allocated to walking, moderate-intensity, and high-intensity physical activities. The time spent sitting reflects the level of sedentary behavior and is evaluated separately. In calculating all physical activities, the criterion was that the activity had been performed continuously for at least 10 min. Physical activity levels were calculated by multiplying the activity duration (minutes), frequency (days), and MET coefficient (multiple of oxygen consumption at rest) and expressed in “MET-minutes/week” units. In this context, walking was multiplied by a MET value of 3.3, moderate-intensity activities by 4, and high-intensity activities by 8 to determine the score. Statistical analyses were conducted based on the total physical activity level obtained in the study.

### 2.5 Data collection

To collect data, scientific research and ethics committee approval was first obtained from the Ethics Committee of a state university with approval number E-26428519-050.99-129835 dated 13.06.2024. The course schedules of the students planned to be included in the sampling were first reviewed, and the data collection process was planned in detail in advance so as not to affect the course processes. Considering the fatigue of the courses, it was decided to collect data from the students before the courses. For this reason, at least 1 h before the courses, detailed information about the research was presented within the university, and data collection was carried out in the lecture halls by having the participants fill out a voluntary participation consent form. The entire process was conducted in accordance with the latest version of the Helsinki Declaration, and data collection took approximately 35 min. Data were collected in person from participants in October 2024.

### 2.6 Data analysis

Before performing statistical analyses, the structure and suitability of the data set were checked. First, a normality test was applied to the data, and the results indicated that the data could be analyzed using parametric tests. Therefore, Pearson correlation and regression techniques were used in the analyses.

According to the results in Table 2, the normality values of the measurement tools are within the ±1 range, and these results are accepted in the literature as being within the normal distribution (George and Mallery, 2019). The Cronbach’s Alpha coefficients are considered reliable according to Karagöz (2017).

**Table 2:** Results related to measurement tools.

	Factors	Skewness	Kurtosis	Cronbach’s *α*
	Orthorexia nervosa	−0.050	0.181	0.723
Big five personality	Extraversion	0.297	0.833	0.755
	Agreeableness	−0.509	0.947	0.688
	Conscientiousness	−0.272	0.018	0.764
	Emotional stability	0.112	0.181	0.751
	Openness to experience	0.070	−0.212	0.702
	Body image	−0.481	−0.577	0.915

## 3 Results

**Figure 1:**
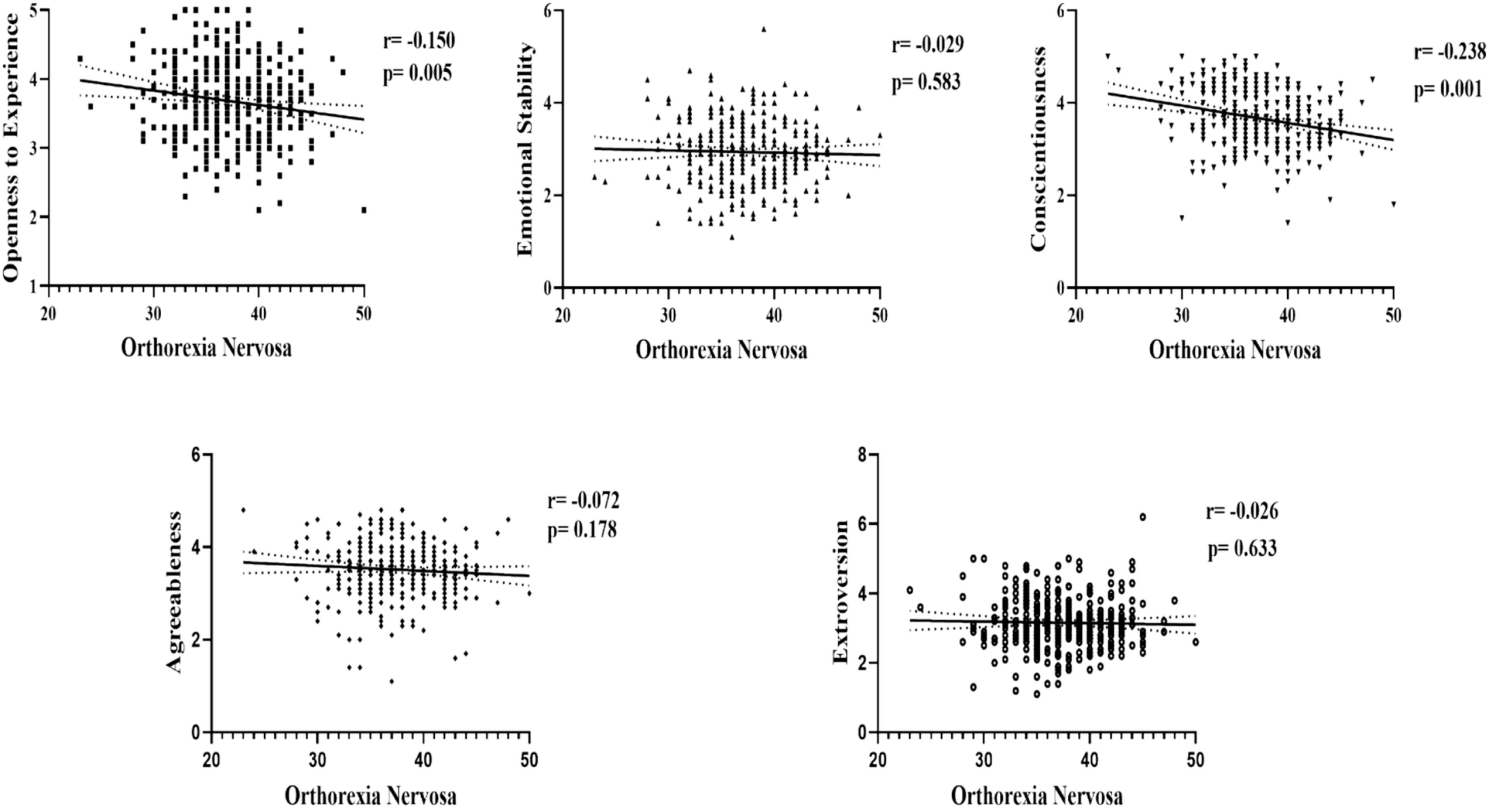
Correlation results between personality traits and orthorexia nervosa.

When interpreting the Pearson correlation analysis in Figure 1, considering that high ORTO-15 scores indicate low orthorexic tendencies, low-level significant negative correlations were observed between orthorexia nervosa and Conscientiousness (*r* = −0.238; *p* = 0.000) and Openness to Experience (*r* = −0.150; *p* = 0.005). On the other hand, orthorexia nervosa showed low levels of significant negative correlations with Extraversion (*r* = −0.026; *p* = 0.633), Agreeableness (*r* = −0.072; *p* = 0.178), and Emotional Stability (*r* = −0.029; *p* = 0.583).

**Figure 2:**
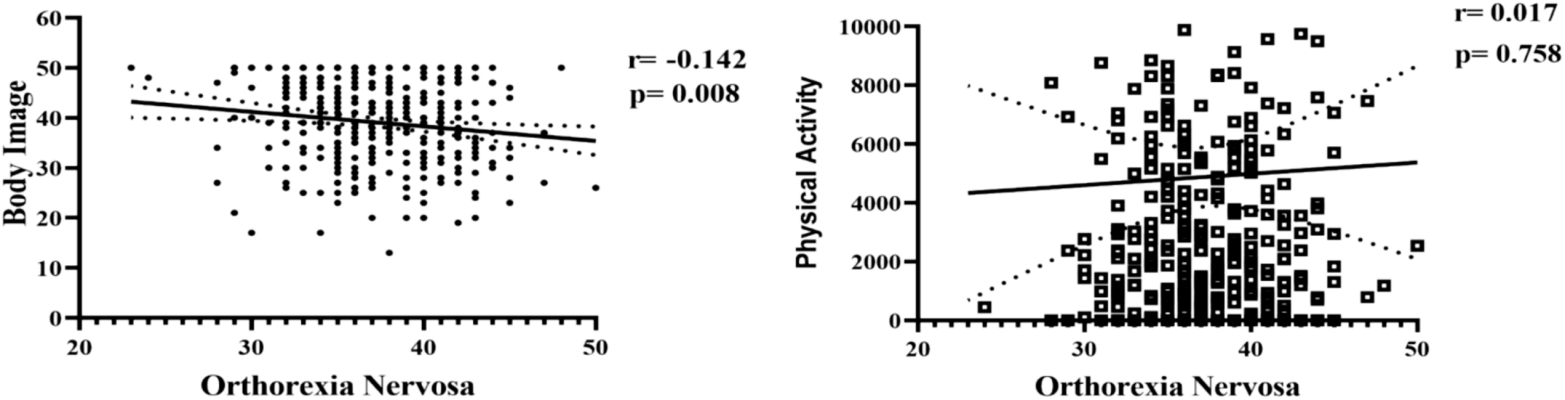
Correlation results between body image, physical activity, and orthorexia nervosa.

According to the results of the Pearson correlation analysis in Figure 2, considering that high ORTO-15 scores represent lower orthorexic tendencies, a low level of significant negative correlation was found between orthorexia nervosa and body image (*r* = −0.142; *p* = 0.008). This finding suggests that individuals with high levels of body satisfaction tend to maintain healthy eating behaviors in a more flexible manner and may be less prone to orthorexic behaviors. In contrast, no significant relationship was found between orthorexia nervosa and physical activity level (*r* = −0.017; *p* = 0.758).

**Figure 3:**
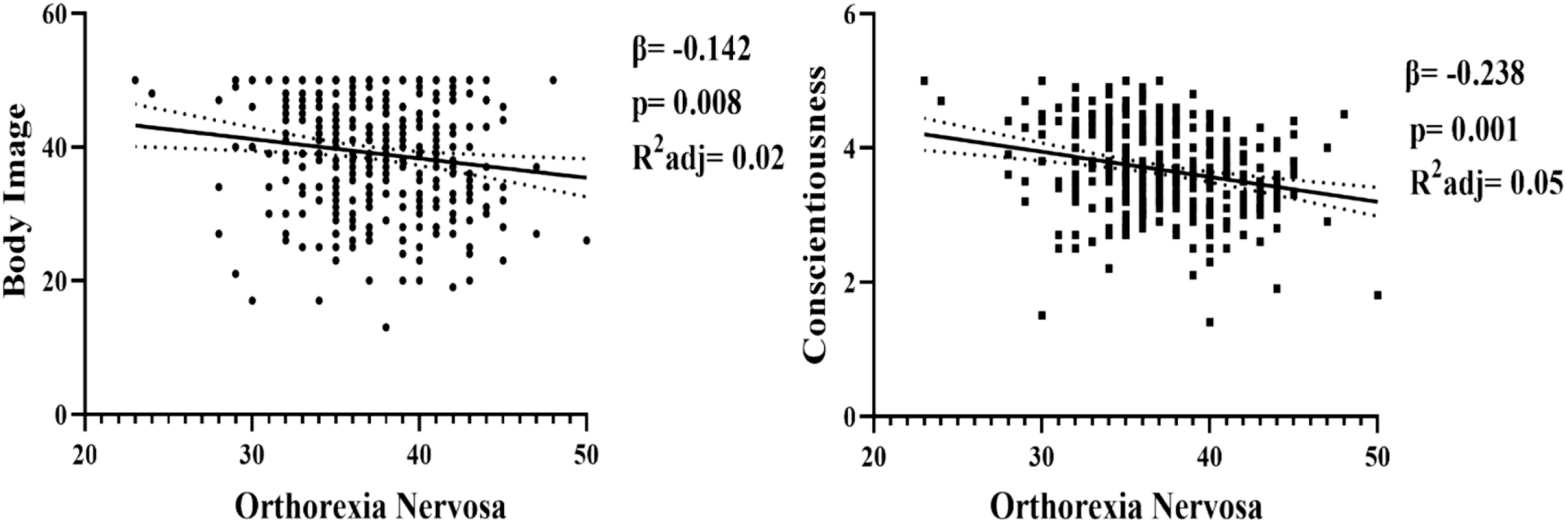
Diagnosis of orthorexia nervosa.

The simple linear regression analysis conducted on the prediction of orthorexia nervosa in Figure 3 revealed that conscientiousness (*β* = −0.238; *t* = −4.563; *p* = 0.000) among personality traits has a significant negative predictive power on orthorexia nervosa. It can be stated that 5% of the variance in orthorexia nervosa is explained by conscientiousness. Another regression result showed that body image (*β* = −0.142; *t* = −2.675; *p* = 0.008) has a negative and significant predictive power on orthorexia nervosa. It can be stated that 2% of the variance in orthorexia nervosa is explained by body image.

## 4 Discussion and conclusion

The aim of this study is to identify orthorexic tendencies in non-clinical adult individuals and to examine the relationship between these tendencies and body image, physical activity level, and the five major personality traits. The findings obtained in the study provide important clues for understanding the effect of individual differences on attitudes toward healthy eating. In particular, the finding that personality traits and body image perception are associated with orthorexic tendencies draws attention to the psychological foundations of eating behaviors. When evaluated in conjunction with existing knowledge in the field, these findings demonstrate that ON is a multidimensional construct that cannot be reduced to mere dietary preferences. In this study, no statistically significant relationship was found between ON and certain personality traits such as extroversion, agreeableness, and emotional stability. This finding suggests that the aforementioned personality dimensions do not directly influence individuals’ rigid and obsessive attitudes toward healthy eating or are insufficient to explain this influence.

The research findings reveal a significant negative correlation between openness to experience and ON scores. Since high ON scores reflect healthy eating behaviors and low scores reflect orthorexic tendencies, this finding suggests that individuals who are open to experience may be more prone to orthorexic attitudes. Although this relationship may seem unexpected at first glance, it makes sense when considering the different aspects of openness to experience. In general, openness to experience reflects an individual’s interest in new experiences, creative thinking, and alternative lifestyles (Tan et al., 2018). However, some studies suggest that this personality trait may lead to a greater search for information, a tendency toward natural nutrition, and individual awareness, particularly in the areas of health and nutrition, which, when combined with a need for control and perfectionism in some individuals, may fuel rigid eating attitudes (Oberle et al., 2017; Brytek-Matera et al., 2019). This situation shows that openness to experience does not always result in flexibility, but can lead to more rule-bound and obsessive eating behaviors in some individuals when combined with excessive internal control. Indeed, ON is characterized by an individual starting out with the goal of healthy eating, but over time, this behavior transforms into rigid rules, food selectivity, and psychological pressure (Koven and Abry, 2015). Individuals who are open to experience, especially those who are more sensitive to healthy living ideologies, may be more likely to engage in selective and rule-bound behaviors regarding eating. Depa et al. (2019) and colleagues emphasize that increased health obsession in individuals open to experience may pave the way for reduced flexibility and obsessive eating attitudes in the name of healthy eating. In this context, a high level of openness to experience may play a role in some individuals’ pursuit of a healthy lifestyle crossing into pathological boundaries.

The findings obtained within the scope of the study show that the conscientiousness factor has a significant negative correlation with ON scores. Considering that low scores in ON represent orthorexic tendencies, this result suggests that individuals with high conscientiousness may exhibit more rigid and control-oriented behaviors in their attitudes toward healthy eating. Conscientiousness is associated with an individual’s ability to regulate internal impulses, develop goal-oriented behaviors, and maintain behavioral stability (Tangney et al., 2004). This construct, which is generally evaluated in relation to psychological well-being, can lead to rigid and restrictive attitudes replacing flexible eating habits in some individuals by integrating with excessive control needs and rigid behavior patterns. In the literature on eating behavior, there is evidence that as conscientiousness levels increase, so do cognitive control over food intake and dietary restrictions (Konttinen et al., 2009). When evaluated within this framework, it is natural for individuals with high conscientiousness to adopt a more disciplined approach toward healthy eating goals; however, this discipline can, in some cases, lead to behavioral rigidity and a loss of psychological flexibility. Various studies have also emphasized that perfectionism, control-orientedness, and excessive sensitivity in food selection, which are among the basic characteristics of orthorexic tendencies, may be related to high conscientiousness (Varga et al., 2013; Pollack and Forbush, 2013). Additionally, it has been noted that conscientiousness may be associated with negative outcomes such as increased mental preoccupation and feelings of guilt in eating behavior (Kalika et al., 2024). In this context, the findings reveal that high conscientiousness does not necessarily lead to harmonious or healthy outcomes in every individual; on the contrary, it may encourage orthorexic attitudes by leading to excessive control of eating behaviors in some individuals. Therefore, the functionality of the concept of conscientiousness can manifest in different ways depending not only on its level but also on the individual’s motivational structure, perception of control, and health-related belief systems.

The research findings revealed a significant negative correlation between body image and scores obtained from the ON scale. When the decrease in scores on the orthorexia scale is interpreted as an increase in orthorexic tendencies, this result indicates that individuals with a higher body image perception are at increased risk of developing rigid, obsessive, and inflexible attitudes toward healthy eating behaviors. Body image is a multidimensional construct encompassing an individual’s evaluations of their physical appearance and the cognitive and emotional processes accompanying these evaluations (Grogan, 2017). Although positive body image is generally associated with psychological well-being in the literature, some studies suggest that individuals with high body awareness may have motivations to maintain and control their external appearance, which can result in food selectivity and excessive health-focused eating behaviors (Mazzeo et al., 2024; Oberle et al., 2017). Especially in situations where appearance-based success and social acceptance are internalized, healthy eating behaviors can become dependent on the individual’s self-worth, which can lead to a structure based on strict norms and moral judgments in eating behaviors (Turner and Lefevre, 2017). In this context, it is possible that high body image satisfaction may combine with the pursuit of a healthy lifestyle in some individuals, leading to an inflexible, idealized eating pattern and reinforcing orthorexic behavior patterns. Recent studies have also shown that appearance-focused attitudes and feelings of control over one’s body are significant predictors of orthorexia nervosa, particularly among young adults (Elias et al., 2022; Martinovic et al., 2022). Therefore, the findings suggest that body image perception should not be evaluated solely as a protective factor; when combined with appearance-centered health motivations, it may create a tendency to excessively control eating behaviors in individuals, thereby laying the groundwork for the emergence of orthorexic attitudes.

This study aims to contribute to the literature by examining the effect of individuals’ personality traits and body image perceptions on orthorexia nervosa tendencies. The findings show that conscientiousness, openness to experience, and body image variables are significantly related to orthorexic attitudes. According to the findings, as conscientiousness and openness to experience levels increase, orthorexic behaviors also increase. Similarly, it was found that orthorexic tendencies were more pronounced in individuals with high body image satisfaction. These findings suggest that individual differences may play a decisive role not only in healthy lifestyle choices but also in the evolution of these choices to a pathological level. On the other hand, no statistically significant relationship was found between orthorexia nervosa and certain variables such as extroversion, agreeableness, emotional stability, and physical activity level. This suggests that these variables may be related to orthorexia nervosa through indirect pathways or different contextual factors rather than through direct effects. In conclusion, it can be said that orthorexia nervosa tendencies have a multidimensional structure and develop in interaction with the individual’s personality traits, body image, and health-related value systems. While the findings of this study provide important relational trends, they also contain some methodological limitations. Firstly, the fact that only quantitative data was used limits the in-depth understanding of the psychosocial dynamics underlying the participants’ orthorexic attitudes. In addition, the fact that the data was collected through self-reporting should be carefully evaluated in terms of cognitive bias and social desirability effects. Furthermore, the fact that the study sample belongs to a specific age and socio-cultural group may limit the generalizability of the findings to different demographic profiles. Therefore, adopting mixed-method approaches and comparing different cultural contexts in future research will contribute to obtaining more comprehensive and in-depth findings. The use of only basic statistical analyses (Pearson correlation and univariate regression) in the study is a methodological limitation arising from the limited sample size. This situation can be improved in future studies by using multivariate models. Another limitation of this study is the use of the ORTO-15 scale. Although it is a frequently preferred tool for assessing orthorexic tendencies, it has been criticized in the literature for its structural Emotional stability and low reliability. The use of more valid and reliable measurement tools is recommended in future research.

## Data Availability

The raw data supporting the conclusions of this article will be made available by the authors, without undue reservation.
